# Producing Cyclopropane Fatty Acid in Plant Leafy Biomass *via* Expression of Bacterial and Plant Cyclopropane Fatty Acid Synthases

**DOI:** 10.3389/fpls.2020.00030

**Published:** 2020-02-07

**Authors:** Shoko Okada, Matthew Taylor, Xue-Rong Zhou, Fatima Naim, David Marshall, Stephen J. Blanksby, Surinder P. Singh, Craig C. Wood

**Affiliations:** ^1^ CSIRO Land and Water, Canberra, ACT, Australia; ^2^ CSIRO Agriculture and Food, Canberra, ACT, Australia; ^3^ Center for Crop Disease Management, Faculty of Science and Engineering, School of Molecular and Life Sciences, Curtin University, Perth, WA, Australia; ^4^ Central Analytical Research Facility, Institute for Future Environments, Queensland University of Technology, Brisbane, QLD, Australia

**Keywords:** dihydrosterculic acid, oleochemical, branched chain fatty acid, cyclopropane fatty acid synthase, *Nicotiana benthamiana*, triacylglycerol

## Abstract

Saturated mid-chain branched fatty acids (SMCBFAs) are widely used in the petrochemical industry for their high oxidative stability and low melting temperature. Dihydrosterculic acid (DHSA) is a cyclopropane fatty acid (CPA) that can be converted to SMCBFA *via* hydrogenation, and therefore oils rich in DHSA are a potential feedstock for SMCBFA. Recent attempts to produce DHSA in seed oil by recombinant expression of cyclopropane fatty acid synthases (CPFASes) resulted in decreased oil content and poor germination or low DHSA accumulation. Here we explored the potential for plant vegetative tissue to produce DHSA by transiently expressing CPFAS enzymes in leaf. When CPFASes from plant and bacterial origin were transiently expressed in *Nicotiana benthamiana* leaf, it accumulated up to 1 and 3.7% DHSA in total fatty acid methyl ester (FAME), respectively, which increased up to 4.8 and 11.8%, respectively, when the *N. benthamiana* endogenous oleoyl desaturase was silenced using RNA interference (RNAi). Bacterial CPFAS expression produced a novel fatty acid with a cyclopropane ring and two carbon-carbon double bonds, which was not seen with plant CPFAS expression. We also observed a small but significant additive effect on DHSA accumulation when both plant and bacterial CPFASes were co-expressed, possibly due to activity upon different oleoyl substrates within the plant cell. Lipidomics analyses found that CPFAS expression increased triacylglycerol (TAG) accumulation relative to controls and that DHSA was distributed across a range of lipid species, including diacylglycerol and galactolipids. DHSA and the novel CPA were present in phosphatidylethanolamine when bacterial CPFAS was expressed in leaf. Finally, when plant diacylglycerol acyltransferase was coexpressed with the CPFASes DHSA accumulated up to 15% in TAG. This study shows that leaves can readily produce and accumulate DHSA in leaf oil. Our findings are discussed in line with current knowledge in leaf oil production for a possible route to DHSA production in vegetative tissue.

## Introduction

**Figure 1:**
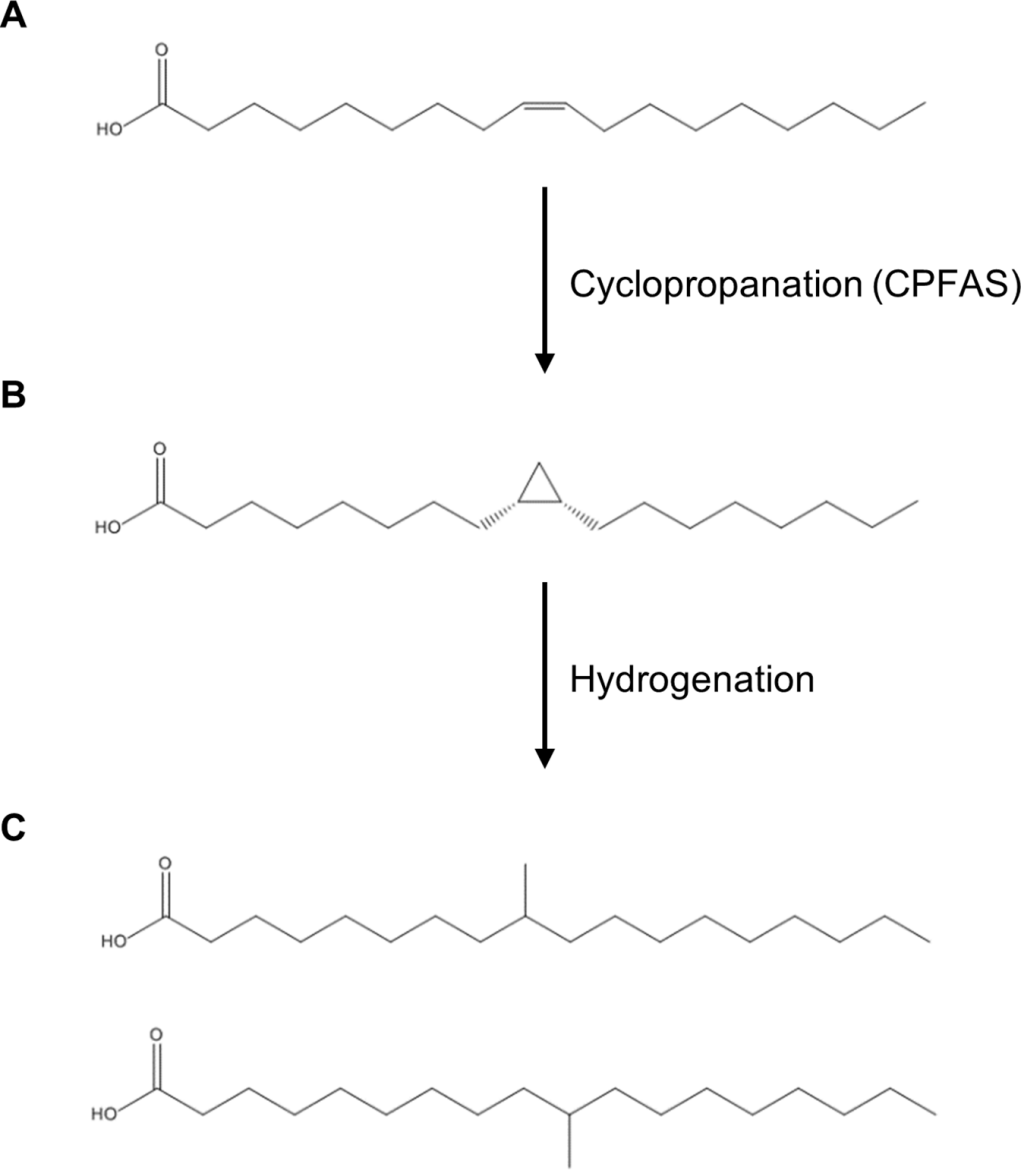
Chemical structures of fatty acids and corresponding reactions relevant to this study. **(A)** oleic acid; **(B)** dihydrosterculic acid; **(C)** isostearic acid isomers.

Saturated mid-chain branched fatty acids (SMCBFAs) offer unique properties that combine the benefits of saturated and unsaturated fatty acids and are thus an important component of the oleochemical industry (Kinsman, 1979; Biermann and Metzger, 2008). SMCBFA have no carbon-carbon bonds, and therefore are less susceptible to free radical attack that provides high oxidative stability compared to unsaturated fatty acids [e.g. oxidative stability at 110°C for soybean oil fatty acid methyl ester (FAME) = 3.1 h, purified saturated branched-chain FAME = 64 h; (Ngo et al., 2011)]. On the other hand, the mid-chain methyl groups confer a lower melting point over saturated fatty acids of similar chain lengths (e.g. stearic acid Tm = 69.3°C, tuberculostearic acid Tm = 20–26°C; Schmidt and Shirley, 1949; Haynes, 2016; **Figure 1**). Compared to either monounsaturated fatty acids or saturated fatty acids, SMCBFAs have high oxidative stability with lubricity at low temperatures, features that are sought after in many oleochemicals (Kinsman, 1979; Zhang et al., 2004; Ngo et al., 2011; Hasselberg and Behr, 2016). One such example of SMCBFA is tuberculostearic acid, where the methyl group is found midway along the fatty acyl chain (**Figure 1**). Currently isostearic acids such as tuberculostearic acid are chemically synthesized using purified fatty acids (Kinsman, 1979; Biermann and Metzger, 2008), however such chemical synthesis methods are expensive and require extra steps of purification to remove undesired isomers (Lou et al., 2004) or are relatively inefficient (Palko et al., 2013).

A potentially elegant and alternative method of synthesis of mid-chain branched fatty acids is *via* the *in planta* production of cyclopropane oils that can be extracted and hydrogenated in post-harvest processing to yield SMCBFA products (**Figure 1**). Cyclopropane fatty acids are widely found in bacteria, trypanosomatids, Myriapoda, and higher plants (reviewed in Oudejans et al., 1971; Fish et al., 1981; Sebedio and Grandgirard, 1989; Grogan and Cronan, 1997), and in this report we focus on the production of dihydrosterculic acid (DHSA), a naturally occurring fatty acid with a mid-chain cyclopropane ring across the C9–C10 carbons (cis-9,10-methyleneoctadecanoic acid; DHSA; **Figure 1B**). The biochemical and molecular bases for the biosynthesis of cyclopropane fatty acids has been elucidated in various higher plants (Bao et al., 2002; Yu et al., 2011) and bacteria (Wang et al., 1992). The biological function of cyclopropane fatty acids is proposed to be one related to stress response and adaptation of membranes to changes in pH, salt, drought, and high temperature stress (reviewed in Knivett and Cullen, 1965; Kuiper and Stuiver, 1972; Grogan and Cronan, 1997; Shabala and Ross, 2008). The general class of cyclopropane fatty acid synthases (CPFASes) operate on monounsaturated acyl chains on phospholipids to produce cyclopropane rings. The *Escherichia coli* (Ec) CPFAS is proposed to cyclopropanate C16:1 and C18:1 that are predominantly esterified to the sn-2 position of phospholipids (Hildebrand and Law, 1964), whereas in plants the CPFAS cyclopropanates the oleoyl chain on the *sn*-1 position of phosphatidylcholine (PC, Bao et al., 2003).

There are limited reports of transgenic expression of CPFAS in plants. Expression of EcCPFAS produced DHSA in tobacco leaf calli accumulating up to approximately 3% of total fatty acids (Schmid, 1995). Tobacco suspension cells were used for the expression of *Sterculia foetida* CPFAS demonstrating accumulation of 4% DHSA in total fatty acids (Bao et al., 2002). In seeds the cotton (Gh) CPFAS expressed in a high oleic background of *fad2/fae1 Arabidopsis* accumulated approximately 1% DHSA in mature seed lipids, despite having potential access to a high level of oleic acid as substrate (Yu et al., 2011). In contrast EcCPFAS in the same seed context produced up to 9% DHSA, and up to 35% with additional expression of *S. foetida* lysophosphatidic acid acyltransferase (Yu et al., 2014; Yu et al., 2018), suggesting that one barrier to DHSA synthesis and accumulation in transgenic seed oil may be due to differences in the site of synthesis on PC. Despite the relatively high accumulation of DHSA in these developing *Arabidopsis* seeds it was found that seeds with greater than 9% DHSA suffered poor germination rates, and over 40% of the DHSA was found to accumulate on PC. When EcCPFAS was coexpressed with a lipid handling enzyme in *Camelina* seed, DHSA accumulated only up to 6% and still over 30% of DHSA remained in polar lipids (Yu et al., 2019). Collectively these results indicate that DHSA is produced on polar lipids in developing seeds, however the channeling of DHSA from polar lipids into seed oil is not efficient (Yu et al., 2018), or it affects seed quality and successive germination in higher amounts of DHSA (Yu et al., 2014). Like DHSA production in transgenic seed, similar problems with unusual fatty acids being accumulated on membrane lipids and adversely affecting oil content and germination has been found in transgenic seed engineered to produce ricinoleic acid (van Erp et al., 2011) and epoxy fatty acid (Li et al., 2012).

Although oilseeds are generally considered the major source of food oils and feedstocks for oleochemical industries, recent advances in metabolic engineering have opened the possibility of producing high levels of oil in leaf biomass (reviewed in Vanhercke et al., 2019). These advances have seen leaves produce over 30% of dry weight as triacylglycerol (Vanhercke et al., 2017). Given the difficulties in generating commercially viable levels of unusual fatty acids in seeds while avoiding impacts on seed viability and germination, it has been proposed that metabolically engineered oils could be produced in leafy biomass (Wood, 2014). For engineering oils into leaves, transient leaf assays using *Nicotiana benthamiana* have proven helpful in rapidly combining multiple genes into functional pathways (Wood et al., 2009; Vanhercke et al., 2013; Reynolds et al., 2015). The endogenous fluxes of lipids in transient assays have also been modified using endogene silencing techniques that allow improved fluxes of critical substrates into introduced metabolic pathways (Naim et al., 2012).

Here we explore the production and accumulation of DHSA in a transient leaf expression system and monitor the placement of this unusual fatty acid on a range of lipid species including those in membranes and oils. In order to achieve higher DHSA production we rely upon the silencing of endogenous oleoyl desaturases to raise oleic levels in leaves using an RNA silencing technique that does not interfere with transgene expression (Naim et al., 2012). Although both plant and bacterial CPFASes produce DHSA that accumulates into leaf oil we found that both genes were capable of accumulating DHSA in oil to a maximum of 15%. Furthermore, we also found that expression of EcCPFAS increased the relative oil content in leaves. Based on the findings from this study we discuss possible implications for the production and accumulation of DHSA in plant vegetative tissue.

## Results

### Cotton and *Escherichia coli* Cyclopropane Fatty Acid Synthases Expressed in *Nicotiana benthamiana* Leaf Produce Dihydrosterculic Acid

**Figure 2:**
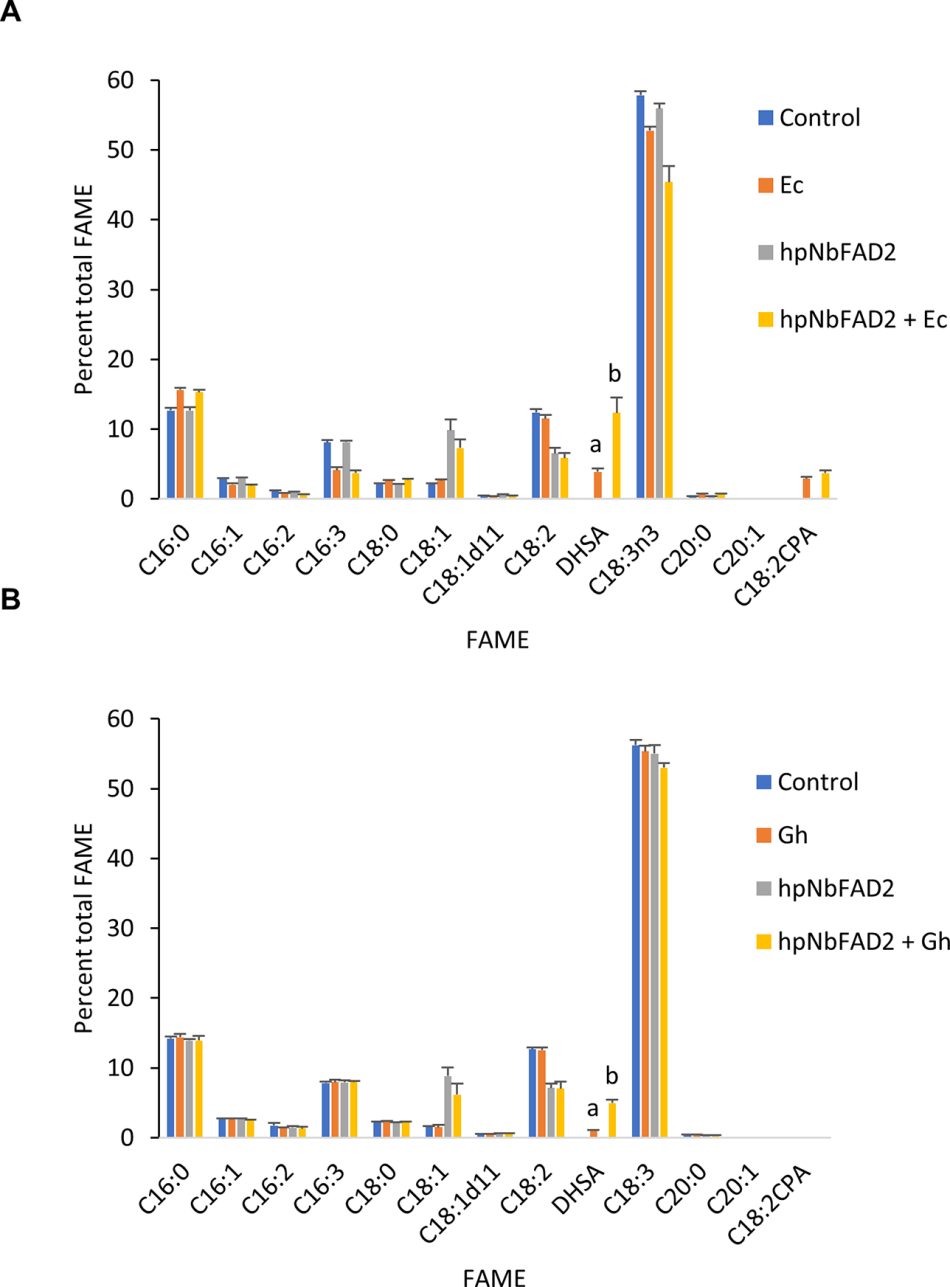
Cyclopropane fatty acid (CPA) production in *Nicotiana benthamiana* leaf transiently expressing cyclopropane fatty acid synthase and increasing oleic acid by silencing the endogenous oleoyl desaturase (NbFAD2) increases CPA accumulation. **(A)** Total fatty acid methyl ester (FAME) profile of *N. benthamiana* leaf total lipid extracts expressing cotton cyclopropane fatty acid synthase (GhCPFAS), tomato yellow leaf curl virus suppressor protein V2, green fluorescent protein (GFP), and *hpNbFAD2.1*. **(B)** FAME profile of *N. benthamiana* leaf total lipid extracts expressing *Escherichia coli* cyclopropane fatty acid synthase (EcCPFAS), V2, GFP, and *hpNbFAD2.1*. Error bars are standard deviations of six and seven biological replicates for the GhCPFAS and EcCPFAS set, respectively. Different letters above the bars indicate significant differences (p < 0.01). V2, tomato yellow leaf curl virus viral suppressor; GFP, green fluorescent protein; hp, hairpin.

We first explored the feasibility of producing dihydrosterculic acid in plant leaf using the *N. benthamiana* leaf transient expression system (Wood et al., 2009). Two CPFASes from *E. coli* (EcCPFAS) and cotton (GhCPFAS) were tested, which produced 3.7 ± 0.4% (n = 7) and 0.9 ± 0.1% (n = 6) DHSA in total FAME, respectively (**Figure 2**). The DHSA peak was confirmed by gas chromatography-mass spectrometry (GC-MS) against a reference standard dihydrosterculic (DHS) methyl ester. We then increased the oleic acid content in *N. benthamiana* to see if increased substrate availability would assist in higher levels of DHSA production. To do this we used a hairpin construct to silence the endogenous oleoyl desaturase (NbFAD2) *via* RNA interference (RNAi), herein termed *hpNbFAD2.1*, which was previously shown to increase total oleic acid content in the leaf transient expression system by six-fold compared to the control when tomato yellow leaf curl virus suppressor protein V2 was coexpressed (Naim et al., 2012). When *hpNbFAD2.1* was coexpressed with Ec- or GhCPFAS production of DHSA significantly increased three- and five-fold to 11.8 ± 2.1% (n = 7, T test t(7) = −9.9, p < 0.01) and 4.8 ± 0.4% (n = 6, T test t(6) = −18.9, p < 0.01) in total FAME, respectively, compared to when the CPFASes were expressed without *hpNbFAD2.1* (**Figure 2**).

**Figure 3:**
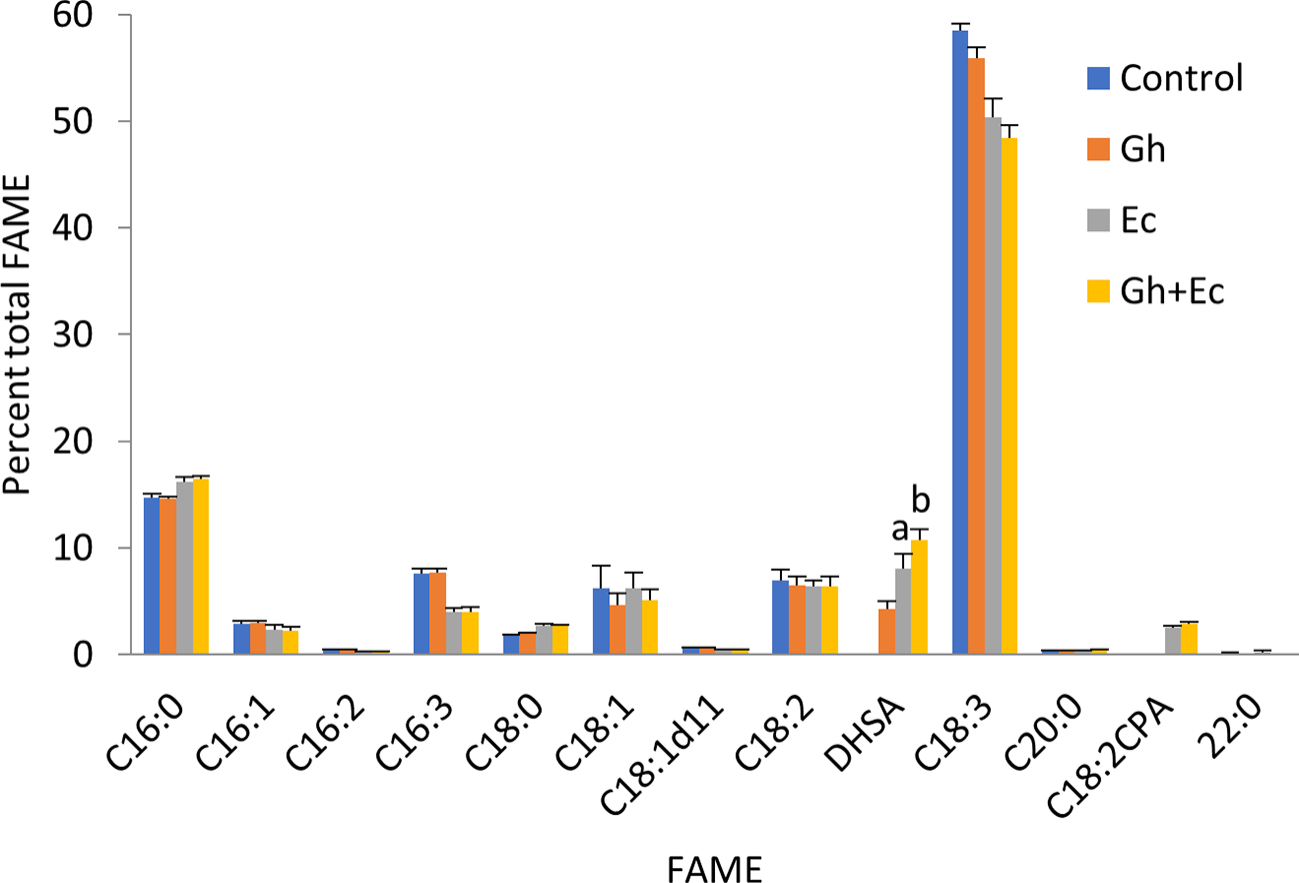
Coexpressing cotton and *Escherichia coli* cyclopropane fatty acid synthases (CPFASes) shows additive effect on DHSA accumulation. Total fatty acid methyl ester (FAME) profile of *Nicotiana benthamiana* leaf total lipid extracts expressing cyclopropane fatty acid synthases separately (Gh, Ec) or together (Gh+Ec), each set coexpressed with tomato yellow leaf curl virus suppressor protein V2, green fluorescent protein (GFP), and *hpNbFAD2.1*. The control expression contained V2, GFP, and *hpNbFAD2.1*. Error bars are standard deviations of six biological replicates. Different letters above the bars indicate significant differences (p < 0.01). Gh, cotton CPFAS; Ec, *E. coli* CPFAS.

Next, we expressed Ec- and GhCPFAS individually and in combination in the hpNbFAD2.1 background to see the effect of co-expressing the two CPFASes on DHSA accumulation in *N. benthamiana* leaf (**Figure 3**). In this particular experiment Gh- or EcCPFAS expression alone produced 4.2 ± 0.7% (n = 6) and 8.0 ± 1.3% (n = 6) DHSA in total FAME, respectively, however when these two CPFASes were co-expressed there was a small but significant increase in DHSA accumulation to 10.7 ± 0.9% DHSA compared to EcCPFAS expression alone (n = 6, T test t(10) = −3.9, p < 0.01).

### Structural Investigation of a Novel Cyclopropane Fatty Acid Produced by *Escherichia coli* Cyclopropane Fatty Acid Synthase Expression

**Figure 4:**
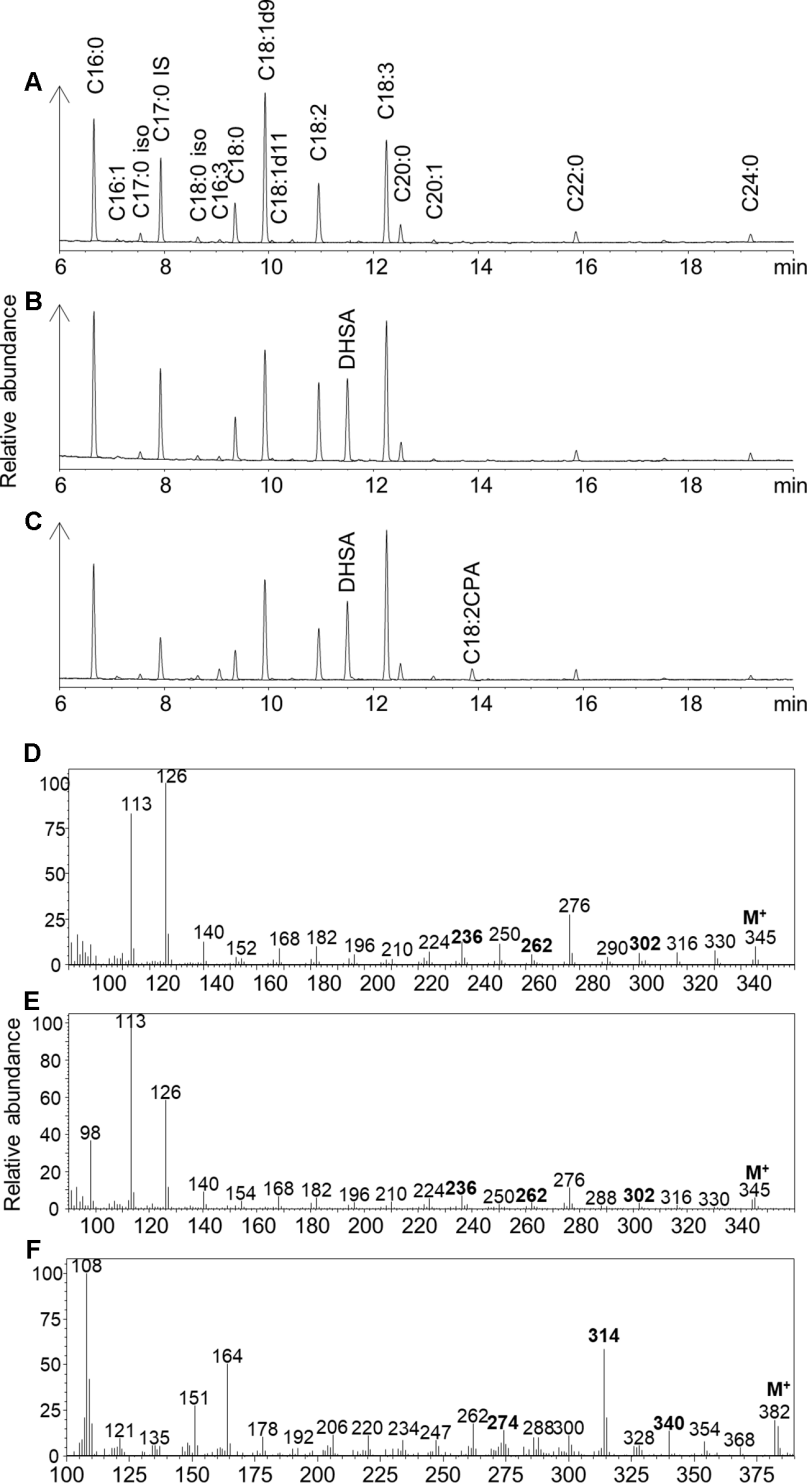
Gas chromatography traces of **(A)** Fatty acid methyl esters (FAMEs) of the triacylglycerol (TAG) fraction of *Nicotiana benthamiana* leaf total lipid extracts expressing tomato yellow leaf curl virus suppressor protein V2, green fluorescent protein (GFP), *hpNbFAD2.1*, and *Arabidopsis thaliana* diacylglycerol acyltransferase (AtDGAT1); iso, isomer; IS, internal standard C17:0. **(B)** FAMEs of the TAG fraction of *N. benthamiana* leaf total lipid extracts expressing tomato yellow leaf curl virus suppressor protein V2, GFP, *hpNbFAD2.1*, AtDGAT1, and cotton cyclopropane fatty acid synthase (GhCPFAS); **(C)** FAMEs of the TAG fraction of *N. benthamiana* leaf total lipid extracts expressing tomato yellow leaf curl virus suppressor protein V2, GFP, *hpNbFAD2.1*, AtDGAT1, and *Escherichia coli* cyclopropane fatty acid synthase (EcCPFAS); **(D)** 4,4-dimethyloxazoline derivative of C18:2CPA; **(E)** pyrrolidine derivative of C18:2CPA; **(F)** 3-pyridylcarbinol derivative of C18:2CPA.

EcCPFAS expression in *N. benthamiana* leaf generated an unidentified compound that was not detected with GhCPFAS expression or in the negative control (**Figure 4**). This compound migrated at 13.879 min in GC analysis of FAMEs of the triacylglycerol (TAG) fraction (1–2%; **Figure 4C**). GC-MS analysis indicated that this compound was a 19-carbon fatty acid containing a cyclopropane ring, with two degrees of unsaturation based on the retention time and *m*/*z* fragmentation pattern. To determine if EcCPFAS can cyclopropanate C18:3Δ9Z,12Z,15Z we expressed the gene in *Saccharomyces cerevisiae* and exogenously supplied it with C18:3. Subsequently, the same compound produced in *N. benthamiana* was detected in the GC trace (**Supplementary Figure 1**). Further GC-MS analysis of this compound with 4,4-dimethyloxazoline, 3-pyridylcarbinol, and pyrrolidine derivatives also agreed with the FAME analysis (**Figures 4D–F**), as having a cyclopropane ring and two degrees of unsaturation. There was evidence from the mass spectra of these derivatives to suggest the location of the carbon-carbon double bonds being Δ12 and Δ15. While a characteristic odd mass (*m*/*z* 247) was observed with the 3-pyridylcarbinol ester indicative of a cyclopropane ring on Δ9-10, the lack of mass abundance and inconsistencies with the characteristic 12 unit mass difference between Δ9 and Δ10 formed during fragmentation of the cyclopropane ring (Zhang et al., 1987) did not allow us to absolutely determine the position of the cyclopropane ring. Similarly, ozone-induced dissociation (OzID; Thomas et al., 2008) analysis tentatively identified this fatty acid to have the cyclopropane ring on Δ9-10, though again this position was not definitive. While further investigation of the structure of this compound is ongoing, we will refer to it as C18:2CPA for the remainder of this paper.

### Lipid Class Analysis of Cyclopropane Fatty Acid-Containing Species

A more extensive analysis of the fluxes of cyclopropane fatty acids in transient leaf assays was conducted using a combination of GC analysis of thin layer chromatography (TLC) fractions and liquid chromatography-tandem mass spectrometry (LC-MSMS) of total lipid extracts from leaves expressing either Gh- or EcCPFAS, *hpNbFAD2.1*, green fluorescent protein (GFP), and V2.

#### Phosphatidylcholine

**Figure 5:**
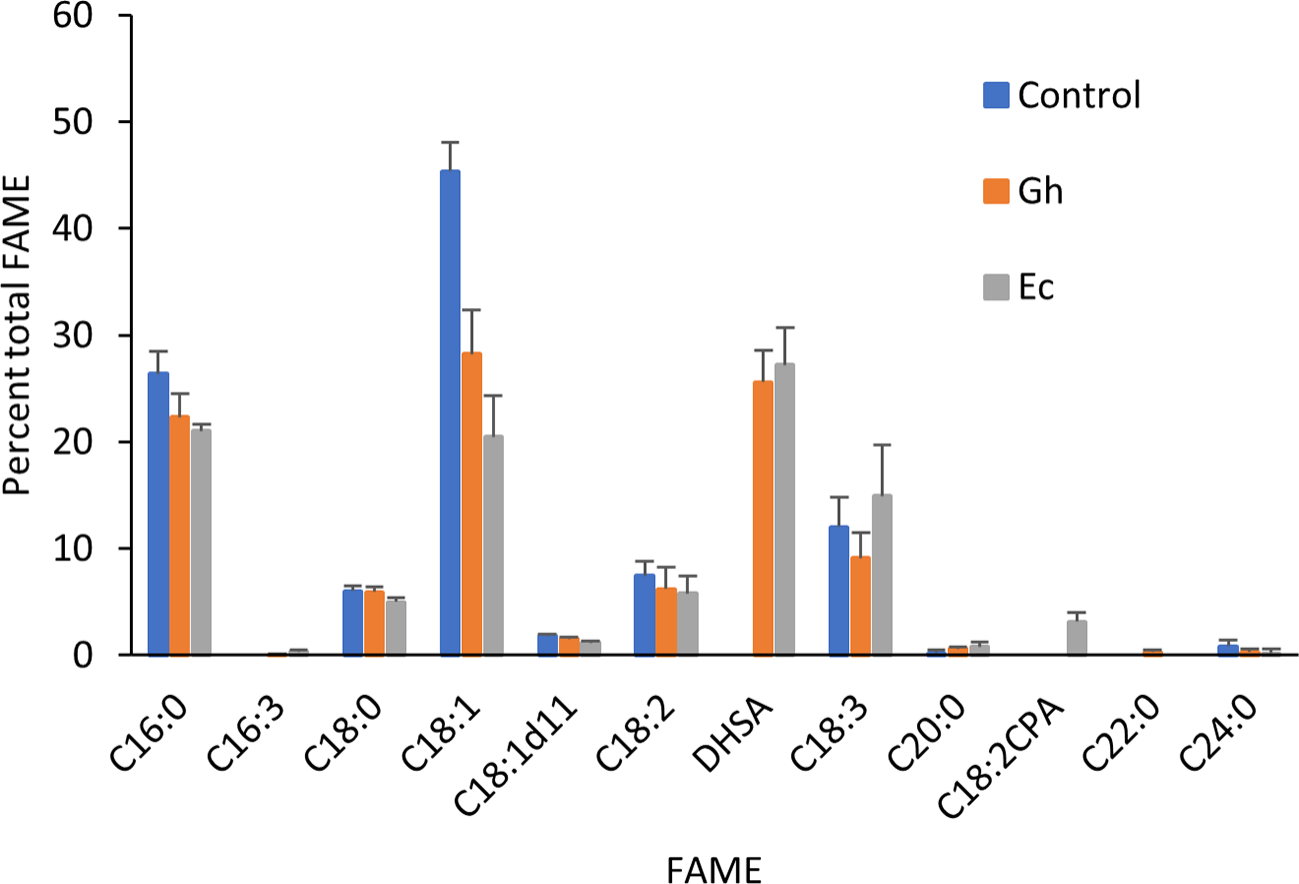
Fatty acid methyl ester (FAME) profile of phosphatidylcholine fraction in *Nicotiana benthamiana* leaf total lipid extracts expressing cotton or *Escherichia coli* cyclopropane fatty acid synthase (CPFAS). Each CPFAS was coexpressed with tomato yellow leaf curl virus suppressor protein V2, green fluorescent protein (GFP), and *hpNbFAD2.1.* The control contained V2, GFP, and *hpNbFAD2.1*. Error bars are standard deviations of six biological replicates. Gh, cotton CPFAS; Ec, *E. coli* CPFAS.

**Figure 6:**
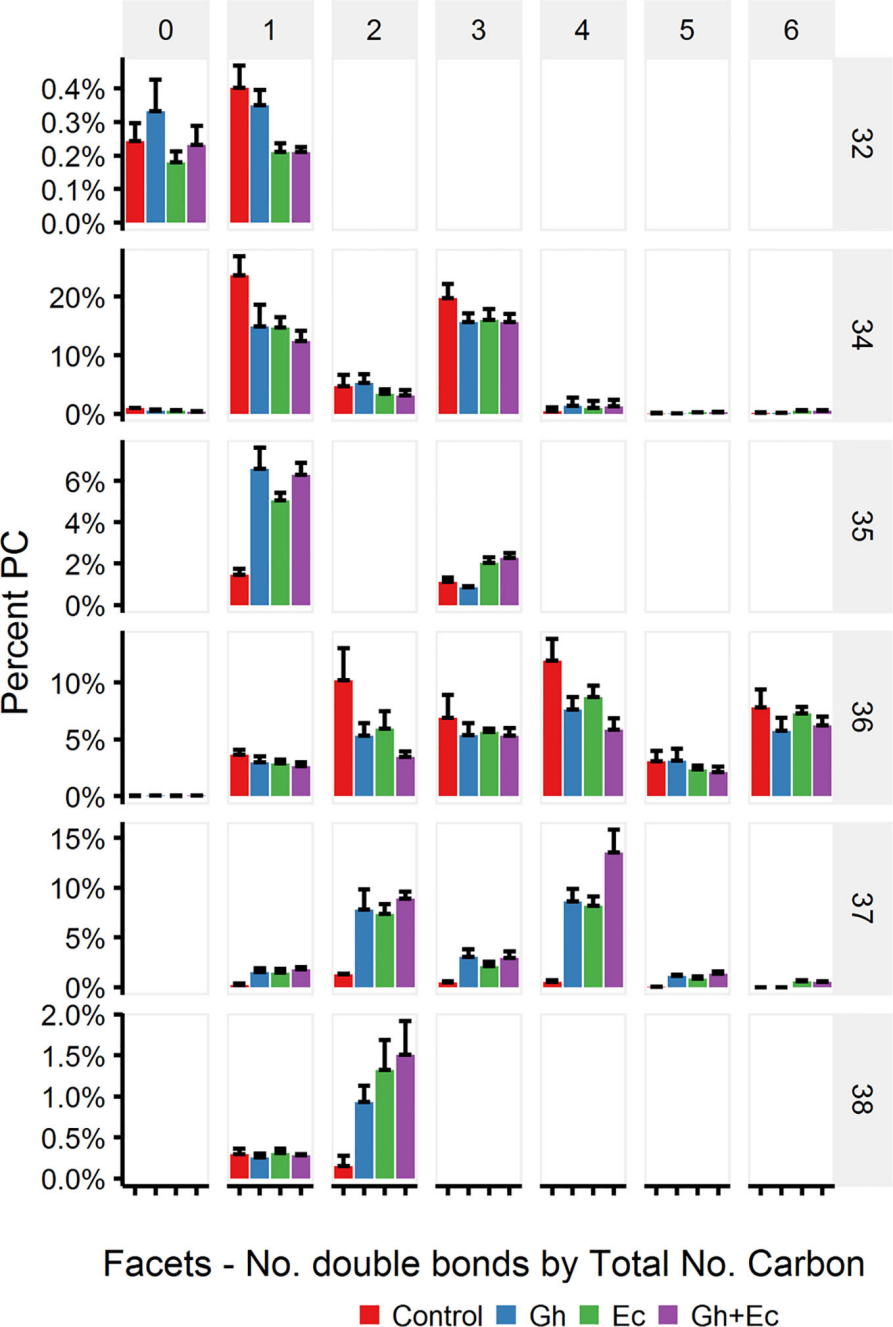
Phosphatidylcholine (PC) species in total lipid extracts of *Nicotiana benthamiana* leaf expressing cotton and/or *Escherichia coli* cyclopropane fatty acid synthase (CPFAS), tomato yellow leaf curl virus suppressor protein V2, green fluorescent protein (GFP), and *hpNbFAD2.1* identified by liquid chromatography-tandem mass spectrometry (LC-MSMS). The control contained V2, GFP, and *hpNbFAD2.1*. Error bars are standard deviations of six biological replicates. Ec, *E. coli* CPFAS; Gh, cotton CPFAS.

Total lipids extracted from leaves expressing either Gh- or EcCPFAS were subjected to TLC for GC analysis of the PC fraction. Both Gh- or EcCPFAS expression resulted in 25–27% accumulation of DHSA in the PC fraction as determined by GC analysis (**Figure 5**). LC-MSMS analysis showed an increase predominantly in the odd chain species PC 35:1, 35:3, 37:2, and 37:4 and also to a lesser extent PC 37:3 and the even numbered PC 38:2 (**Figure 6**). The structural composition of PC species of *N. benthamiana* leaves expressing EcCPFAS were more thoroughly investigated using OzID to elucidate positions of unsaturation, as well as composite tandem mass spectrometry methods employing both collision- and ozone-induced dissociation to identify the fatty acyl chains at each *sn-*position (Thomas et al., 2008; Pham et al., 2014). For the odd-chain PCs of composition PC 35:1 and PC 35:3, OzID mass spectra revealed that these predominantly comprised structures with a C16:0 fatty acid at the *sn-*1 position with either a DHSA or C18:2CPA occupying the *sn*-2 position, respectively. Interestingly, PC 36:2 contained roughly equivalent proportions of C18:1 Δ9 and Δ11. The only other PC species observed to contain C18:1 Δ11 was PC 37:2, which comprised C18:1 and DHSA with no specificity for the *sn-*position. PC 38:2 comprised two DHSA moieties. PC 37:4 contained both *sn-*positional isomers of C18:3/DHSA, however C18:2CPA was not observed in this species (*i.e.*, PC C18:1/C18:2CPA). Similar to PC 37:2, PC 37:6 was found to contain C18:2CPA on either *sn*-position.

#### Phosphatidylethanolamine

**Figure 7:**
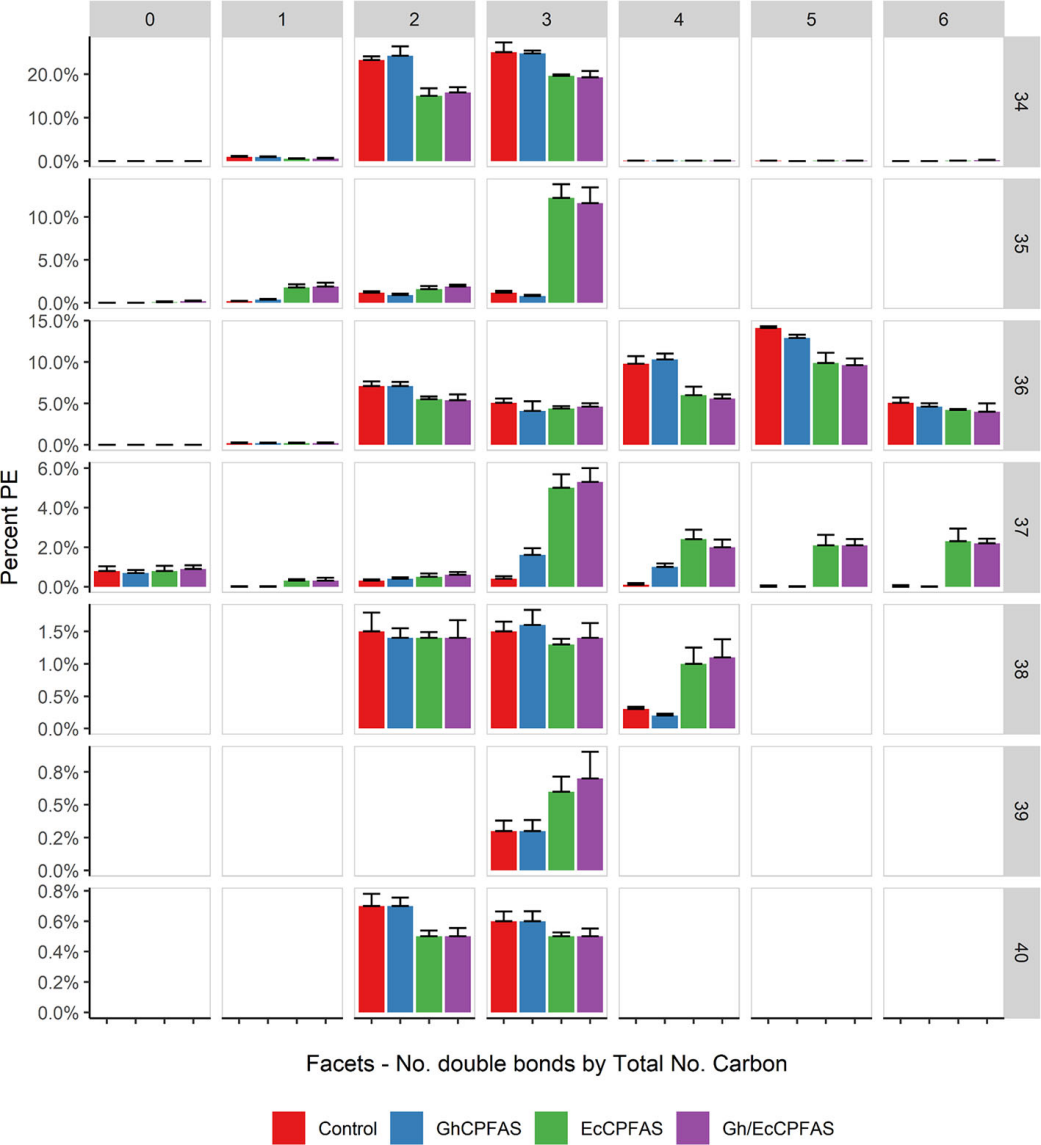
Phosphatidylethanolamine (PE) species in total lipid extracts of *Nicotiana benthamiana* leaf expressing cotton and/or *Escherichia coli* cyclopropane fatty acid synthase (CPFAS), tomato yellow leaf curl virus suppressor protein V2, green fluorescent protein (GFP), and *hpNbFAD2.1* identified by liquid chromatography-tandem mass spectrometry (LC-MSMS). The control contained V2, GFP, and *hpNbFAD2.1*. Error bars are standard deviations of six biological replicates. Ec, *E. coli*; Gh, cotton.

Because EcCPFAS is known to cyclopropanate unsaturated acyl chains bound to phosphatidylethanolamine (PE, Grogan and Cronan, 1997) we investigated the acyl chain composition of PE using LC-MSMS upon Ec- or GhCPFAS expression in *N. benthamiana* leaf (**Figure 7**). Noticeable differences between Ec- and GhCPFAS expression were decrease in PE 34:2 (C16:0/C18:2), PE 34:3 (C16:0/C18:3), PE 36:4 (C18:1/C18:3 and C18:2/C18:2), and PE 36:5 (C18:2/C18:3), and increase in PE 35:1 (C16:0/DHSA), PE 37:3 (C18:2/DHSA), PE 37:4 (C18:3/DHSA and C18:2/C18:2CPA), PE 37:5 (C18:2/C18:2CPA), and PE 37:6 (C18:3/C18:2CPA) when EcCPFAS was expressed (both with or without GhCPFAS) relative to the negative control and GhCPFAS expression. GhCPFAS also produced minor amounts of PE 37:3 and PE 37:4, however EcCPFAS produced relatively more DHSA as well as C18:2CPA-containing PE species. PE 38:4 (DHSA/C18:2CPA) was also detected in leaf expressing EcCPFAS.

#### Diacylglycerol and Triacylglycerol

**Figure 8:**
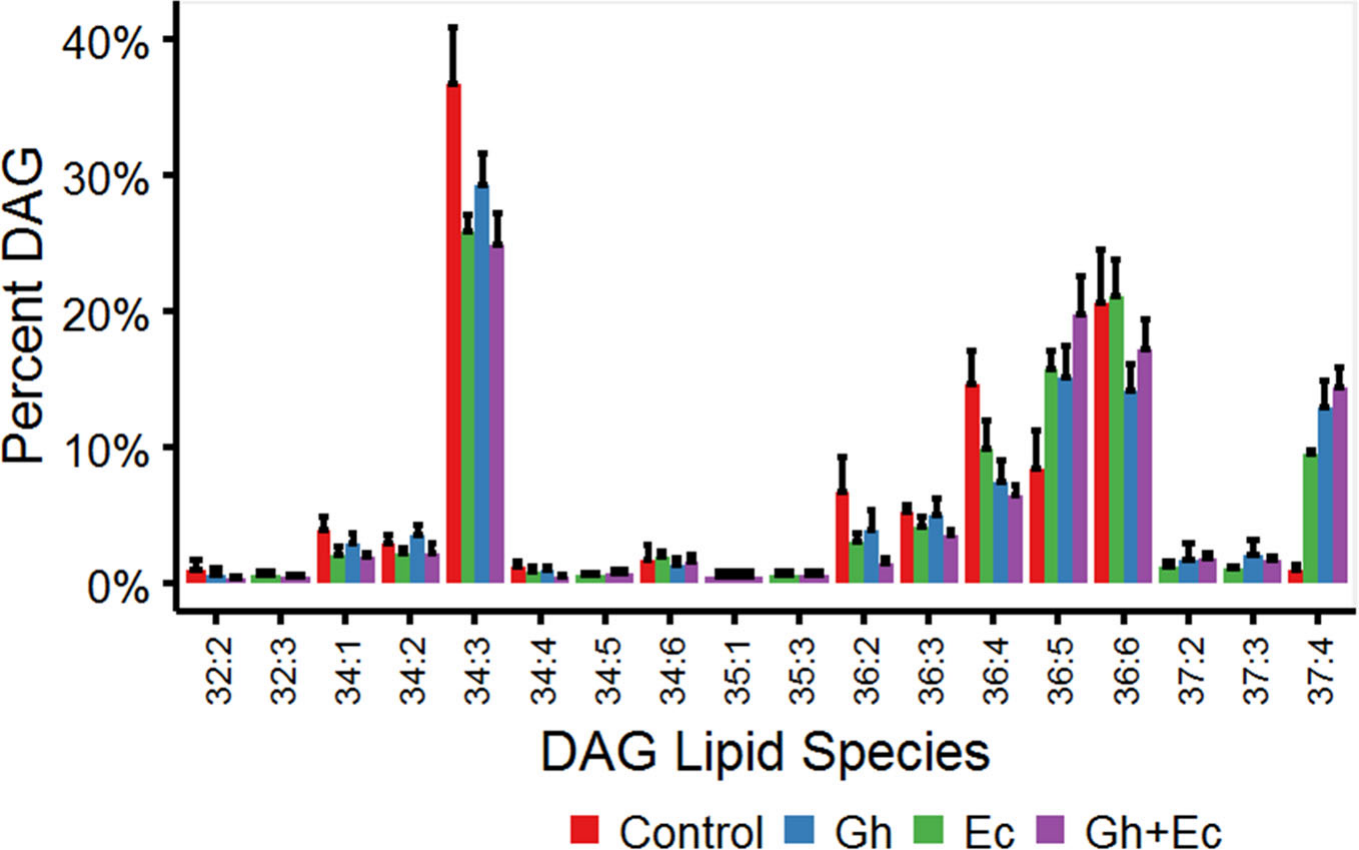
Diacylglycerol (DAG) species in total lipid extracts of *Nicotiana benthamiana* leaf expressing cotton and/or *Escherichia coli* cyclopropane fatty acid synthase (CPFAS), tomato yellow leaf curl virus suppressor protein V2, green fluorescent protein (GFP), and *hpNbFAD2.1* identified by liquid chromatography-tandem mass spectrometry (LC-MSMS). The control contained V2, GFP, and *hpNbFAD2.1*. Error bars are standard deviations of six biological replicates. Ec, *E. coli* CPFAS; Gh, cotton CPFAS.

Diacylglycerol (DAG) and TAG species were analyzed by LC-MSMS to investigate the acyl chain combinations containing DHSA. While many DHSA- containing DAG and TAG species were commonly shared between the two CPFAS-expressing leaf lipid extracts, extracts expressing EcCPFAS contained quantitatively more compared to those with GhCPFAS expression based on the response factors. The most abundant DAG species in *N. benthamiana* transiently expressing either Gh- or EcCPFAS were DAG 34:3 (C16:0/C18:3), DAG 36:6 (C18:3/C18:3), DAG 37:4 (DHSA/C18:3), DAG 36:4 (C18:3/C18:1), and DAG 36:5 (C18:2/C18:3) (**Figure 8**). While other DHSA-containing DAG species were observed [namely, DAG 35:1 (C16:0/DHSA), DAG 37:1 (C18:0/DHSA), and DAG 37:3 (C18:2/DHSA)], DAG 37:4 was approximately 12% of the total DAG species with both Gh- or EcCPFAS expression.

**Figure 9:**
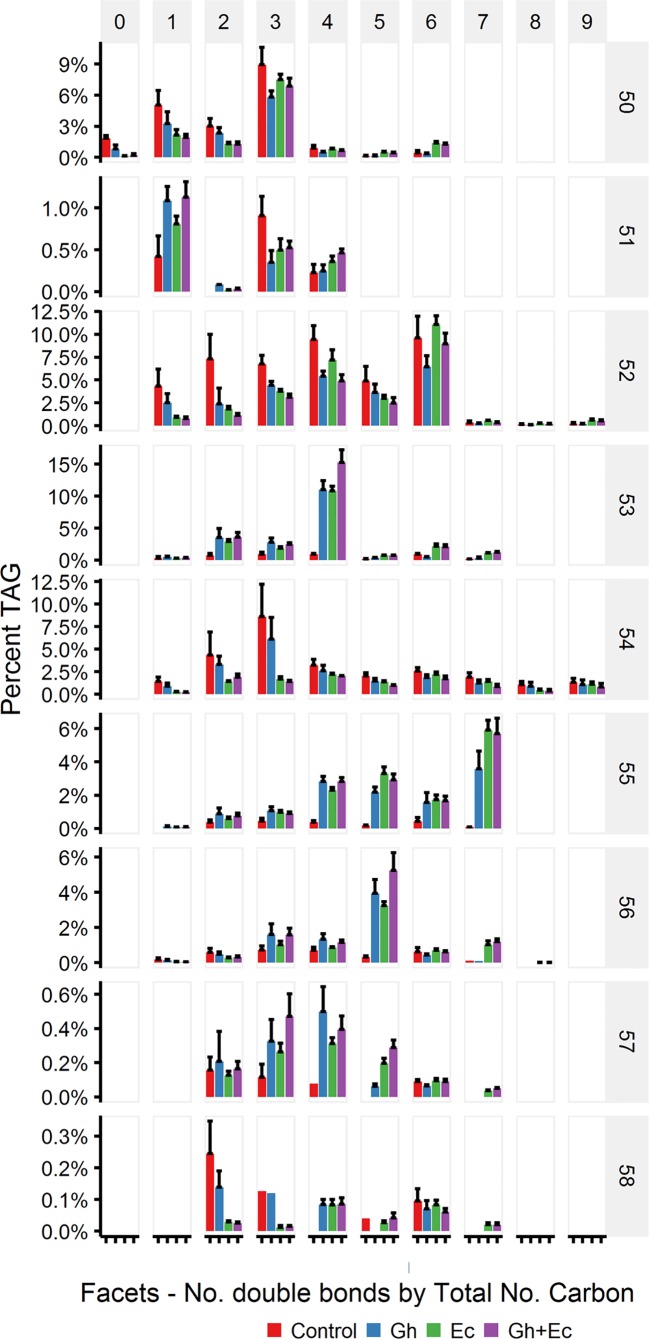
Abundant triacylglycerol (TAG) species containing cyclopropane fatty acids in total lipid extracts of *Nicotiana benthamiana* leaf expressing cotton and/or *Escherichia coli* cyclopropane fatty acid synthase (CPFAS), tomato yellow leaf curl virus suppressor protein V2, green fluorescent protein (GFP), and *hpNbFAD2.1* identified by liquid chromatography-tandem mass spectrometry (LC-MSMS). The control contained V2, GFP, and *hpNbFAD2.1*. Error bars are standard deviations of six biological replicates. Ec, *E. coli* CPFAS; Gh, cotton CPFAS.

The percent distribution of TAG species shows that the most abundant species upregulated with expression of CPFASes are: TAG 53:4, TAG 55:7, TAG 56:5 followed by lower levels of TAG 53:2&3 and TAG 55:4-6. The overall distribution of acyl chains on different TAG species was assessed on triple-quadrupole LC-MS (LC-MS QQQ) by the neutral loss of each acyl chain. The most abundant DHSA- containing TAG species were TAG 53:2 (C16:0/C18:1/DHSA), TAG 53:3 (C16:0/C18:2/DHSA), TAG 53:4 (C16:0/C18:3/DHSA), TAG 55:4 (C18:0/C18:3/DHSA), TAG 55:5 (C18:1/C18:3/DHSA), TAG 55:6 (C18:2/C18:3/DHSA), TAG 55:7 (C18:3/C18:3/DHSA), and TAG 56:5 (DHSA/DHSA/C18:3) (**Figure 9**).

We observed a relative increase in neutral lipids DAG and TAG in total lipids extracted from *N. benthamiana* expressing EcCPFAS, with or without GhCPFAS, compared to those without EcCPFAS expression (**Supplementary Figure 2**).

#### Galactolipids

**Figure 10:**
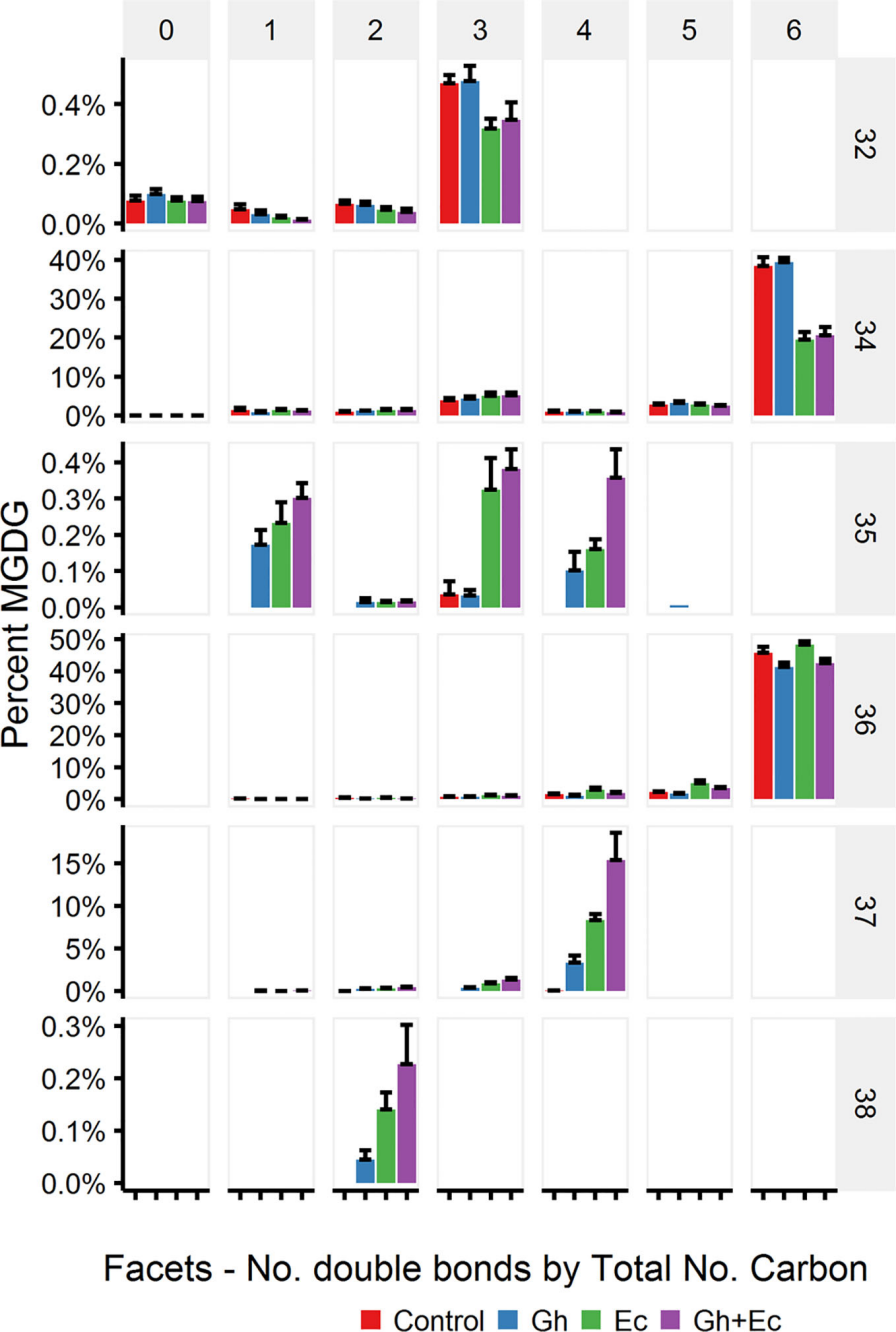
Monogalactosyldiacylglycerol (MGDG) species in total lipid extracts of *Nicotiana benthamiana* leaf expressing cotton and/or *Escherichia coli* cyclopropane fatty acid synthase (CPFAS), tomato yellow leaf curl virus suppressor protein V2, green fluorescent protein (GFP), and *hpNbFAD2.1* identified by liquid chromatography-tandem mass spectrometry (LC-MSMS). The control contained V2, GFP, and *hpNbFAD2.1*. Error bars are standard deviations of six biological replicates. Ec, *E. coli* CPFAS; Gh, cotton CPFAS.

**Figure 11:**
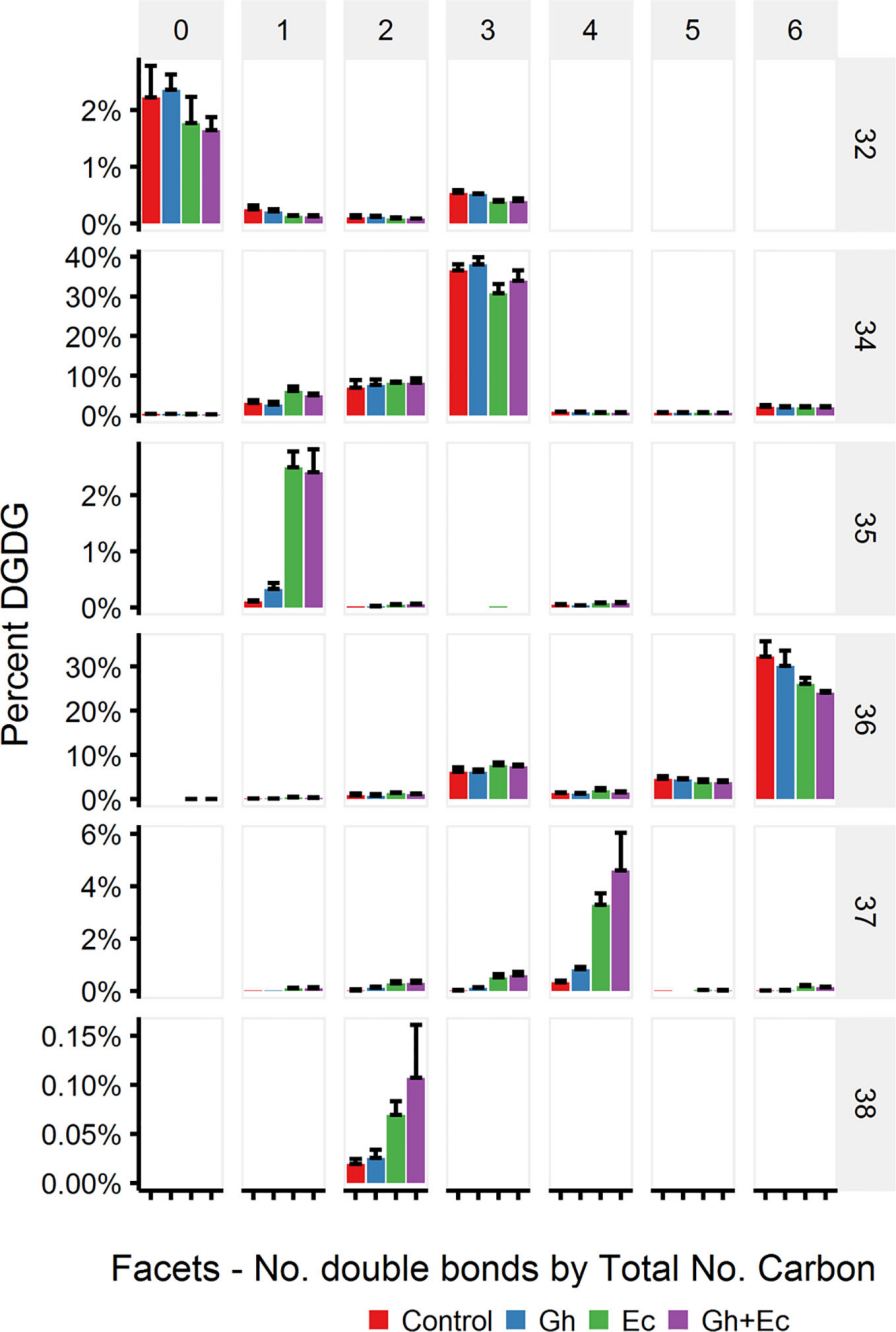
Digalactosyldiacylglycerol (DGDG) species in total lipid extracts of *Nicotiana benthamiana* leaf expressing cotton and/or *Escherichia coli* cyclopropane fatty acid synthase (CPFAS), tomato yellow leaf curl virus suppressor protein V2, green fluorescent protein (GFP), and *hpNbFAD2.1* identified by liquid chromatography-tandem mass spectrometry (LC-MSMS). The control contained V2, GFP, and *hpNbFAD2.1*. Error bars are standard deviations of six biological replicates. Ec, *E. coli* CPFAS; Gh, cotton CPFAS.

LC-MSMS detected only minor amounts of DHSA- containing galactolipid species with CPFAS expression, which were coupled with C18:1, C18:2, or C18:3. The four most common galactolipid species, monogalactosyldiacylglycerol (MGDG) 36:6 (C18:3/C18:3), MGDG 34:6 (C16:3/C18:3), digalactosyldiacylglycerol (DGDG) 34:3 (C16:0/C18:3), and DGDG 36:6 (C18:3/C18:3) accounted for over 80% of each galactolipid species in *N. benthamiana* expressing V2, GFP, and *hpNbFAD2.1*. There was a noticeable decrease in DGDG 34:3 (C16:0/C18:3), DGDG 36:6 (C18:3/C18:3), MGDG 34:6 (C16:3/C18:3), and MGDG 36:6 (C18:3/C18:3) in leaf lipid extracts expressing EcCPFAS, but not those with GhCPFAS (MGDG, **Figure 10**; DGDG, **Figure 11**). With the expression of EcCPFAS MGDG 34:6 was reduced to about 20% of the total MGDG pool. There was a corresponding increase in MGDG 37:4 (DHSA/C18:3) accounting for 15% of the MGDG with the coexpression of both CPFASes, with minor accumulation of MGDG 35:1(C16:0/DHSA), 35:3(C16:0/C18:2CPA), 35:4(C16:3/DHSA), and 38:2(DHSA/DHSA) species.

### Cotton and *Escherichia coli* Cyclopropane Fatty Acid Synthase Expressed in *Nicotiana benthamiana* Leaf Accumulates Dihydrosterculic Acid in Triacylglycerol

**Figure 12:**
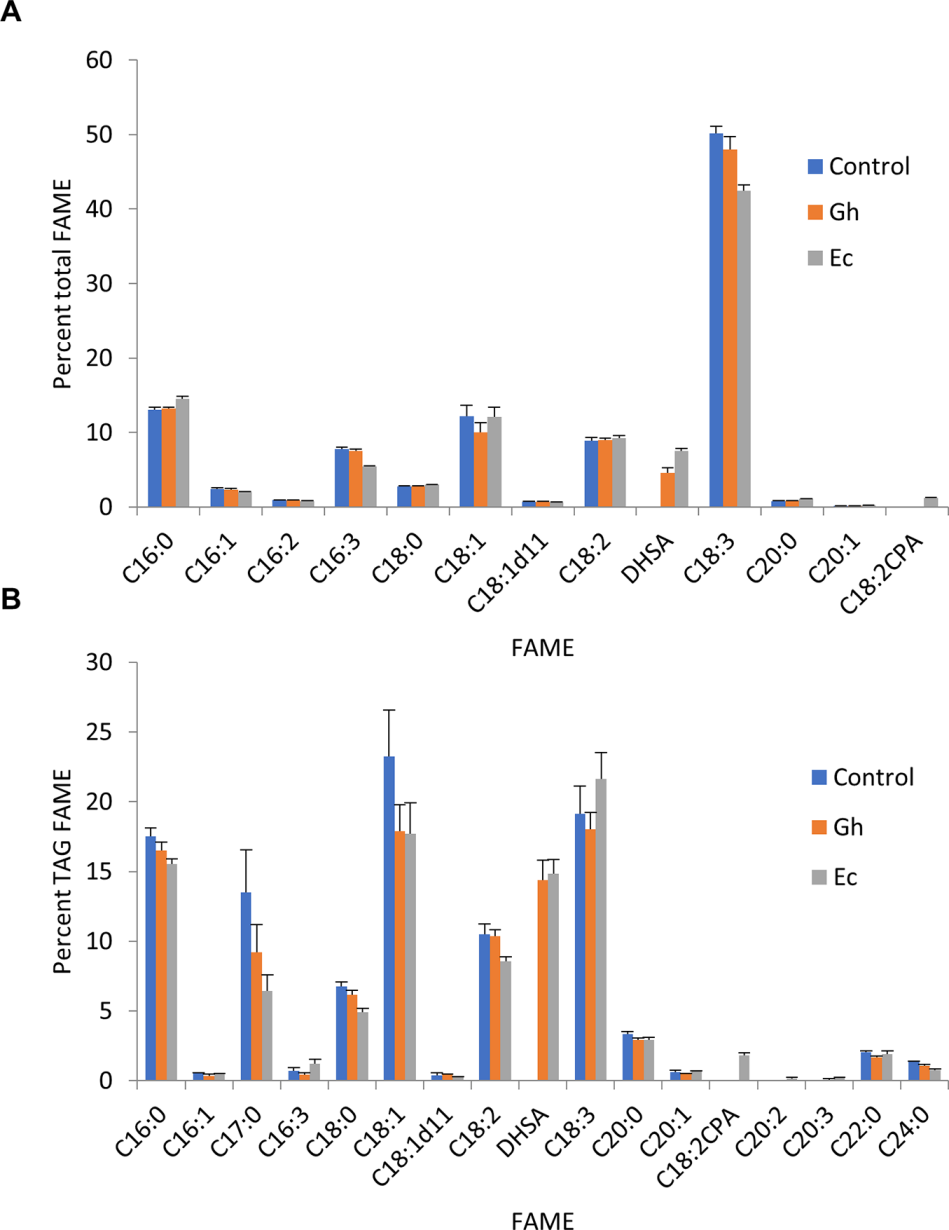
Cyclopropane fatty acid (CPA) accumulates in the triacylglycerol (TAG) fraction in *Nicotiana benthamiana* leaf total lipid extracts expressing cyclopropane fatty acid synthase (CPFAS). **(A)** Total fatty acid methyl ester (FAME) profile of *N. benthamiana* leaf expressing cotton or *Escherichia coli* CPFAS, tomato yellow leaf curl virus suppressor protein V2, green fluorescent protein (GFP), *hpNbFAD2.1*, and *Arabidopsis* diacylglycerol acyltransferase 1 (AtDGAT1). The control contained V2, GFP, *hpNbFAD2.1*, and AtDGAT1. Error bars are standard deviations of six biological replicates. **(B)** FAME profile of the TAG fraction in *N. benthamiana* leaf total lipid extracts expressing cotton or *E. coli* CPFAS, tomato yellow leaf curl virus suppressor protein V2, GFP, *hpNbFAD2.1*, and AtDGAT1. The control contained V2, GFP, *hpNbFAD2.1*, and AtDGAT1. C17:0 FAME was an internal standard added as triheptadecanoin per dry leaf weight prior to extraction. Error bars are standard deviations of six biological replicates. Ec, *E. coli* CPFAS; Gh, cotton CPFAS.

As previously described, it is possible to increase the oil content in transient leaf assays with the expression of *Arabidopsis thaliana* diacylglycerol acyltransferase (AtDGAT1) (Wood et al., 2009). TAG is the lipid class that is currently being used widely throughout the fossil fuel industry as a feedstock for oleochemicals that could be replaced with more sustainable sources of plant oils (Carlsson et al., 2011). TAG can also provide a neutral storage solution for fatty acids such as DHSA that are synthesized on polar lipids such as PC and PE (Yu et al., 2014; Yu et al., 2018; Yu et al., 2019). We coexpressed Gh- or EcCPFAS along with *hpNbFAD2.1*, V2, and AtDGAT1 to see if DHSA could be accumulated in the TAG fraction (**Figure 12B**). In this particular experiment Gh- and EcCPFAS produced DHSA at 4.5 ± 0.7% (n = 6) and 7.5 ± 0.4% (n = 6) in total FAME, respectively (**Figure 12A**), indicating that coexpressing AtDGAT1 with CPFAS did not increase overall DHSA accumulation in *N. benthamiana* leaf.

The FAME profile of TAG fractions from leaf total lipid extracts separated by thin layer chromatography showed that DHSA produced from both Gh- and EcCPFAS accumulated equally in TAG, at approximately 15.8% (GhCPFAS, 15.8 ± 1.3%; EcCPFAS, 15.8 ± 1.1%; n = 6; **Figure 12B**). In all extractions an equal amount of C17:0 TAG (triheptadecanoin) was included to allow monitoring of total TAG content throughout the analysis. Comparison of the relative amount of C17:0 FAME showed that TAG content increased to 1.0 ± 0.2 and 1.5 ± 0.3% leaf dry weight with Gh- and EcCPFAS expression compared to the control without CPFAS expression at 0.7 ± 0.2%, respectively (**Figure 12B**).

## Discussion

One major advantage of transient leaf assays is the ability to rapidly test combinations of different genes and make robust side-by-side comparisons in the context of plant expression. We compared the metabolic flux of DHSA produced by plant- and bacterial-derived CPFAS genes. When expressed individually both enzymes performed similarly in terms of the accumulation of DHSA in TAG. This can be contrasted with similar comparisons in oilseeds where the bacterial CPFAS produced more than five times as much DHSA compared to the plant CPFAS (Yu et al., 2011; Yu et al., 2014). We boosted the oleic acid content of leaves using simultaneous suppression of the endogenous *N. benthamiana FAD2*, although this only results in approximately 10% oleic acid in total lipid. Despite this relatively modest level of substrate, both plant and bacterial CPFASes were capable of a metabolic flux accumulating DHSA to approximately 15% in leaf oils. In comparison developing seed of the *fad2/fae1 Arabidopsis* mutant had close to 80% oleic acid in the polar lipids, yet only accumulated up to 10% CPA in TAG when EcCPFAS was expressed (Yu et al., 2014).

The leaf transient assay format also allowed a streamlined method to combine both the plant and bacterial CPFASes to see if we could further increase DHSA production in leaf. Co-expression of Ec- and GhCPFAS gave the surprising results of a partially additive effect on DHSA accumulation. This result suggests that the CPFAS enzymes are capable of catalyzing different oleoyl substrates. Such a result is consistent with *E. coli* CPFAS acting on oleoyl substrates on both *sn*-1 and -2 positions of PC, and the plant CPFAS operating on the oleoyl chain on the *sn*-1 position of PC (Hildebrand and Law, 1964; Bao et al., 2003; Yu et al., 2014), as well as *E. coli* CPFAS being able to access the oleoyl chain on PE more effectively due to it being the natural substrate in the bacteria (this study; Grogan and Cronan, 1997; Yu et al., 2018). In terms of the variation in the level of DHSA accumulation seen between different experimental sets a review on *N. benthamiana* transient expression by Sheludko (2008) illustrates the large variation in protein expression using this system. This variation seems to occur for many reasons, such as batch-to-batch differences in the physiological state and age of the plants and condition of *Agrobacterium* upon infiltration and during expression, as well as the position of the leaf within an individual plant that is used for expression. In our study, and in other studies looking at modification of fatty acids *via* expression of a lipid modification enzyme there is another layer of variation in the lipid composition between plant batches (commented in Vanhercke et al., 2019), which may also contribute to the variation seen in the level of DHSA accumulation between infiltration sets. Despite this variation we still did observe a consistent increase in DHSA accumulation within batches when the bacterial and cotton CPFASes were coexpressed.

The movement of DHSA from the site of synthesis (PC) into DHSA rich oils can be boosted by the addition of specific DHSA handling enzymes. In plant seed the flux of oleic acid into DHSA could be significantly boosted by the further addition of a DHSA-specific lysophosphatidic acid acyltransferase (SfLPAT2, Yu et al., 2014), or phosphatidylcholine:diacylglycerol cholinephosphotransferase (Yu et al., 2019). We tested co-expression of EcCPFAS and SfLPAT2 to see if leaf tissue could benefit from DHSA channeling to the sn-2 position of polar and neutral lipids, however we did not observe any increase in DHSA accumulation. Our result is in contrast with that reported in *Arabidopsis* seed coexpressing EcCPFAS and SfLPAT2 (**Supplementary Figure 3**; Yu et al., 2014). Further investigation on lipid handling enzymes that will increase DHSA accumulation into leaf oil is currently underway.

Lipidomic analysis of leaves expressing CPFASes found an increase in TAG species that were composed of acyl chains that were derived from thylakoid galactolipid species. Transfer of galactolipids into TAG in leaves has also been observed in senescing leaves of *A. thaliana* (Kaup et al., 2002), ozone-fumigated spinach leaves (Sakaki et al., 1990a), and drought-stressed cotton leaves (El-Hafid et al., 1989). Much of this acyl chain transfer is thought to occur *via* transfer of acyl chains from MGDG to TAG *via* various intermediates such as acyl-CoA and DAG (Sakaki et al., 1990a; Sakaki et al., 1990b; Xiao et al., 2010). As production of DHSA from CPFAS expression resulted in the transfer of galactolipids into TAG, it is possible that DHSA induces a stress response, which in turn induces transfer of the fatty acid into a neutral environment for the leaf in the form of oil. In terms of the recipient DAG species for TAG assembly with DHSA the increase in DAG 36:5 percentage seems to be mainly offset by a decrease in DAG 34:3 percentage in CPFAS expression lines. DAG 34:3 seems to be preferentially used by the endogenous TAG assembly machinery in combination with DHSA (TAG 53:4) when compared to that of DAG 36:5 (TAG 55:6). Therefore, if the ratio of assembly of the different DAG species is consistent but usage as substrate for TAG assembly is different with DHSA then it could possibly explain the relative increase in DAG 36:5 and simultaneous decrease in DAG 34:3.

Interestingly we identified for the first time a novel lipid compound generated by *E. coli* CPFAS, C18:2CPA, which is possibly generated from α-linolenic acid as substrate. We attempted to identify the structure of C18:2CPA using three different derivatization methods for GC-MS analysis, as well as OzID analysis. While we confirmed the positions of the two double bonds on Δ12 and Δ15 from all of the above analyzes, the position of the cyclopropane ring could not be conclusively determined. Like DHSA, C18:2CPA is capable of being hydrogenated to form SMCBFA, and therefore its production is noteworthy from an oleochemical feedstock point of view. This catalytic plasticity of *E. coli* CPFAS (that was not seen in the cotton CPFAS) has the potential to provide various forms of cyclopropane fatty acid as substrates for production of a wide range of SMCBFAs. Another interesting observation to note is the lack of lactobacillic acid (11R,12S-methylene-octadecanoic acid) that could potentially be produced by *E. coli* CPFAS expression in these transient leaf assays. OzID analysis of PC species detected C18:1Δ11 as an acyl chain, which is the endogenous substrate for CPFAS in *E. coli*, from which lactobacillic acid is produced (Grogan and Cronan, 1984). We did not detect any lactobacillic acid in any of our lipid analyzes, which suggests that the C18:1Δ11 is not available to the recombinant *E. coli* CPFAS for cyclopropanation. C18:1Δ11 is proposed to be synthesized by elongation of C16:1Δ9 in the plastid (Xue et al., 2013), and therefore most of it may be on PC in the plastid where it is not accessible to *E. coli* CPFAS.

In transient leaf assays the expression of *E. coli* CPFAS, and presumably the production of DHSA, increased the relative amount of TAG. This is in contrast with oilseed production systems, in which it is commonly reported that production of high levels of unusual fatty acids has negative impacts on the total amount of TAG, including DHSA (Yu et al., 2014), ricinoleic acid (van Erp et al., 2011), and gamma linolenic acid in safflower (Nykiforuk et al., 2012). In oilseeds these bottlenecks in flux can be ascribed to the accumulation of the unusual fatty acid in the cell membranes such as in PC lipids. In our analyses we found that DHSA was present in PC at approximately 25%, which also could benefit from additional lipid handling enzymes that preferentially transfers cyclopropane fatty acids from polar lipids to neutral lipids, i.e. TAG. The sensing of fatty acids in plant cells is a little-known area, however it has been hypothesized that oleic acid is a signaling molecule (Andre et al., 2012). Perhaps accumulation of DHSA at the expense of oleic acid in the tissue interrupts this signaling process allowing more oil to accumulate than is otherwise possible with common fatty acids. Vanhercke et al. (2017) saw a trade-off between carbohydrate (i.e. starch and soluble sugars) and TAG accumulation when genes involved in seed oil synthesis were expressed in *Nicotiana tabacum* leaf. However, the level of PC did not change with TAG accumulation when two of the seed oil synthesis genes were transiently expressed in *N. benthamiana* (Vanhercke et al., 2013). It would be of interest to see where the carbon for increased DHSA accumulation is coming from in our study, which will guide future efforts in generating stable transformants. More detailed investigations will be needed to investigate the molecular basis of TAG increase upon expression of *E. coli* CPFAS in leaf tissue. In conclusion the production of cyclopropane fatty acids in plants could provide a potential feedstock for the production of saturated mid-chain branched fatty acids that are in demand for oleochemical products. Focus on improved movement of DHSA from PC into neutral lipids in further investigations could improve upon the yields achieved thus far. We are currently exploring the combination of DHSA accumulation pathways with the latest iterations of platforms producing high oil in vegetative tissue (Vanhercke et al., 2014).

## Material and Methods

### *Escherichia coli* Cyclopropane Fatty Acid Synthase

The full-length gene coding for EcCPFAS was amplified from DH10B genomic DNA. The nucleotide sequence was 100% identical to accession M98330.

### Cotton Cyclopropane Fatty Acid Synthase

The full-length gene coding for GhCPFAS (accession AY574036) was synthesized (Thermo Fisher Scientific, MA, US) with two silent nucleotide substitutions G2043A and G2551A from the published sequence to remove XhoI restriction sites.

### Construction of 35S Cyclopropane Fatty Acid Synthase Vectors for Plant Expression

pENTR11 (Thermo Fisher Scientific, MA, US) was digested with NcoI, overhangs removed with DNA polymerase I, Large (Klenow) fragment, and re-ligated to generate pENTR-NcoI. *E. coli* and cotton CPFAS genes were cloned into pENTR-NcoI *via* two EcoRI restriction sites and EcoRI and XhoI restriction sites respectively, and subsequently cloned into a cauliflower mosaic virus (CaMV) 35S promoter-driven over-expression vector pXZP393 (Zhou et al., 2013a) using LR Clonase™ II enzyme mix (Thermo Fisher Scientific, MA, US) according to the manufacturer's instructions. Resulting plasmids containing the two CPFASes were used to transform *Agrobacterium tumefaciens* strain AGL1 for *N. benthamiana* leaf transient expression.

### *Nicotiana benthamiana* Leaf Transient Expression

Recombinant AGL1 containing *35S::EcCPFAS* and *35S::GhCPFAS*, viral suppressor *35S::V2*, visual expression marker *35S::GFP*, *N. benthamiana* microsomal oleate desaturase silencer *35S::hpNbFAD2.1*, and *A. thaliana* diacylglycerol acyltransferase *35S::AtDGAT1* (Naim et al., 2012) were grown in Luria-Bertani (LB) broth with appropriate antibiotics for 2 days at 28°C with shaking. On the third day of incubation acetosyringone was added to the cultures to a final concentration of 100 μM and further incubated for 2–3 h, after which the cultures were collected by centrifugation at 2000*g* for 10 min, and resuspended in infiltration buffer (5 mM 2-(*N*-morpholino)ethanesulfonic acid (MES), 5 mM MgSO_4_, 500 μM acetosyringone). Infiltration mixes were prepared with each AGL1 at 0.3 OD600 units and infiltrated using a syringe on the underside of 5–6-week-old *N. benthamiana* leaves. Biological replicates of each experiment were produced by individual agroinfiltration spots on different leaves across different plants. Infiltrated plants were left at 24°C with 10:14 light:dark cycle for 5 days. On the fifth day expression of the infiltrated region was confirmed by presence of the GFP signal, which was harvested, freeze dried overnight, weight recorded, and stored in −80°C until analysis.

### Fatty Acid Methyl Ester Analysis

Total lipid was extracted from leaf tissue as previously described (Naim et al., 2012), with triheptadecanoin (1 µg/mg dry leaf weight, Nu-Chek Prep Inc., MN, US) added to each sample prior to extraction. Lipid extractions equivalent to approximately 2.5 mg dry leaf weight were transmethylated with 0.1 M sodium methoxide in methanol:chloroform = 9:1 at 95°C for 60 min to prepare FAMEs and extracted with hexane. For triacylglycerol analysis extractions equivalent to approximately 10 mg dry leaf weight were separated by TLC on pre-coated silica gel aluminum foils (Fluka, Sigma Aldrich, MO, US) with hexane:diethyl ether:acetic acid = 70:30:1 (vol:vol:vol) as the separation solvent. The TLC foil was briefly stained with iodine and corresponding fractions parallel to the triolein standard were collected, and FAMEs prepared as described above. Total lipid fractionation for phosphatidylcholine analysis was performed according to Wood et al. (2009). GC was performed as previously described (Zhou et al., 2011) using an Agilent 6890N GC (Agilent Technologies, CA, US) equipped with a SGE BPX70 column (Trajan Scientific and Medical Pty Ltd, VIC, Australia, 30 m x 0.25 mm i.d., 0.25 μm film thickness), a flame ionization detector, a split/splitless injector, and an Agilent Technologies 7683 Series autosampler and injector using helium as the carrier gas. Peak responses were calibrated against FAMEs of authentic Nu-Chek gas–liquid chromatography (GLC) standard-411 (Nu-Chek Prep Inc, MN, US), which contains equal-weight amounts of 31 different FAMEs, with added additional equal-weight amount of methyl dihydrosterculate (Matreya LLC, PA, US). FAME peaks were integrated using the Agilent ChemStation software (Agilent Technologies, CA, US), and the percentage was calculated with responses normalized against the corresponding weight of each FAME peak. TAG content as % per dry leaf weight was calculated as per Vanhercke et al., 2013.

GC-MS was performed to confirm the identity of FAMEs not present in the base control infiltration and was carried out on a Shimadzu GC-MS QP2010 Plus ion-trap fitted with on-column injection (Shimadzu Corp., Kyoto, Japan) as previously described (Zhou et al., 2013b), equipped with a SGE BPX70 column (Trajan Scientific and Medical Pty Ltd, VIC, Australia, 30 m x 0.25 mm i.d., 0.25 μm film thickness). Pyrrolidine derivatives were prepared from FAME according to Andersson and Holman (1974). Picolinyl derivatives were prepared directly from FAME according to the method of Destaillats and Angers (2002). 4,4-dimethyloxazoline, pyrrolidine, and picolinyl derivatives were analyzed on the GC-MS as per Zhou et al. (2013b), except for pyrrolidine and picolinyl derivatives, for which an extension of the final holding temperature at 240°C was employed, resulting in a total run time of 35 min. Mass spectra were acquired and processed with GC-MS solution software version 2.61 (Shimadzu Corp., Kyoto, Japan).

### Lipid Class Analysis

LC-MSMS analysis of total lipid extracts was conducted based on previously described methods (Reynolds et al., 2015). Briefly, total lipid extracts were analyzed on an Agilent 6490 triple quadrupole mass spectrometer with jetstream and ion funnel technology (Agilent Technologies, CA, US). Lipid species were separated by liquid chromatography on a Waters (MA, US) BEH C8 (100 mm × 2.1 mm × 2.7 µm) column using an Agilent 1290 LC system (Agilent Technologies, CA, US). Solvents used for the separation of lipid species were mobile phase A (acetonitrile:water, 9:1, with 10 mM ammonium acetate and 0.2% acetic acid) and B (2-isopropanol:acetonitrile:water, 80:15:5, with 10 mM ammonium acetate and 0.2% acetic acid) with a flow rate of 0.2 ml/min. An initial gradient of 1% B was held for 2 min, then raised to 20% B over 3 min to elute PC, PE, DAG, DGDG, and MGDG species followed by a sharp increase to 60% B over 3 min and slowly increased to 70% B for separation of the TAG species over 4 min before re-equilibration at 1% B. The mass spectrometer gas temperature and flow were set to 250°C and 14 L/min and 250°C at 11 L/min for the sheath gas, nebulizer at 45 psi, capillary voltage at 3000 V, and nozzle voltage at 1000 V. The ammonium adducts of MGDG, DGDG, DAG, and TAG species were analyzed by the neutral loss of fatty acid C16 to C20, and the pronated PCs and PEs were identified by a characteristic fragment ion at *m*/*z* 184 or neutral loss of m/z 141, respectively. Multiple reaction monitoring lists were based on the loss of observed fatty acids using a collision of 28 V for all lipid species except DAG where 14 V was used. Chromatograms were integrated using Agilent Quantitative Analysis software version 6.0 (Agilent Technologies, CA, US). Statistical and graphical analysis was conducted using R (R Core Team, 2014) and data packages dplyr and tidyr (Wickham, 2014) and the graphical interpretation using ggplot2 (Wickham, 2010) using Rstudio (www.rstudio.com).

### Ozone-Induced Dissociation Analysis of Total Lipid Extracts of *Nicotiana benthamiana* Expressing *Escherichia coli* Cyclopropane Fatty Acid Synthase

Total lipid extracts of *N. benthamiana* expressing *35S::EcCPFAS*, *35S::V2*, *35S::GFP*, *35S::hpNbFAD2.1*, and *35S::AtDGAT1* were subjected to OzID analysis (Thomas et al., 2008) and composite collision-induced dissociation (CID) and OzID (Marshall et al., 2016) on a modified LTQ XL ion trap mass spectrometer (Thermo Fisher Scientific, MA, US). PC species were separated on a Waters (MA, US) C18 CSH (100 mm × 2.1 mm × 1.7 µm) column, using an initial mobile phase of 12:5% A (water) with 87.5% B (60:40 methanol:acetonitrile) at a flow rate of 0.2 ml/min for 2 min, before ramping to 100% B over 15 min. The post-column eluent was combined by tee infusion with a 0.5 mM solution of sodium acetate or lithium acetate to facilitate the exclusive formation of sodium (or lithium) adduct ions by electrospray ionization. PC metal adduct ions were held in the ion trap in the presence of ozone for 100–1000 ms.

### *Saccharomyces cerevisiae* Expression of *Escherichia coli* Cyclopropane Fatty Acid Synthase

EcCPFAS in pENTR-NcoI was used to transfer EcCPFAS into pYES-DEST52 (Thermo Fisher Scientific, MA, US) using LR Clonase™ II enzyme mix (Thermo Fisher Scientific, MA, US) according to the manufacturer's instructions. The resulting plasmid was used to transform *S. cerevisiae* strain INVSc1 (Thermo Fisher Scientific, MA, US) with the Yeast Transformation Kit (Sigma Aldrich, MO, US). Exogenous feeding of yeast, FAME preparation, and GC-MS analysis followed the methods of Zhou et al. (2013b).

## Data Availability Statement

The raw data supporting the conclusions of this article will be made available by the authors, without undue reservation, to any qualified researcher.

## Author Contributions

SO, SS, and CW conceived the project and designed the experiments. SO, MT, X-RZ, FN, DM, and SB conducted the experiments. All authors contributed to writing the manuscript.

## Funding

This project was co-funded by CSIRO and Grains Research and Development Corporation. Access to the Central Analytical Research Facility, operated by the Institute for Future Environments, Queensland University of Technology, was supported by generous funding from the QUT Science and Engineering Faculty.

## Conflict of Interest

The authors declare that the research was conducted in the absence of any commercial or financial relationships that could be construed as a potential conflict of interest.

## Abbreviations

At, *Arabidopsis thaliana*; CPA, cyclopropane fatty acid; CPFAS, cyclopropane fatty acid synthase; DAG, diacylglycerol; DGDG, digalactosyldiacylglycerol; DGAT1, diacylglycerol acyltransferase 1; DHSA, dihydrosterculic acid; Ec, *Escherichia coli*; FAD2, fatty acid desaturase 2; FAME, fatty acid methyl ester; GC, gas chromatography; Gh, *Gossypium hirsutum*; LC, liquid chromatography; MGDG, monogalactosyldiacylglycerol; MS, mass spectrometry; Nb, *Nicotiana benthamiana*; PC, phosphatidylcholine; PE, phosphatidylethanolamine; SMCBFA, saturated mid-chain branched fatty acid; TAG, triacylglycerol; TLC, thin layer chromatography.
