# Supplementary material for: Producing Cyclopropane Fatty Acid in Plant Leafy Biomass via Expression of Bacterial and Plant Cyclopropane Fatty Acid Synthases
Source: Front Plant Sci. 2020 Feb 7;11:30. doi: 10.3389/fpls.2020.00030 (PMC7020751; doi:10.3389/fpls.2020.00030)
Supplement: Supplementary file 1 [file DataSheet_1.pdf]

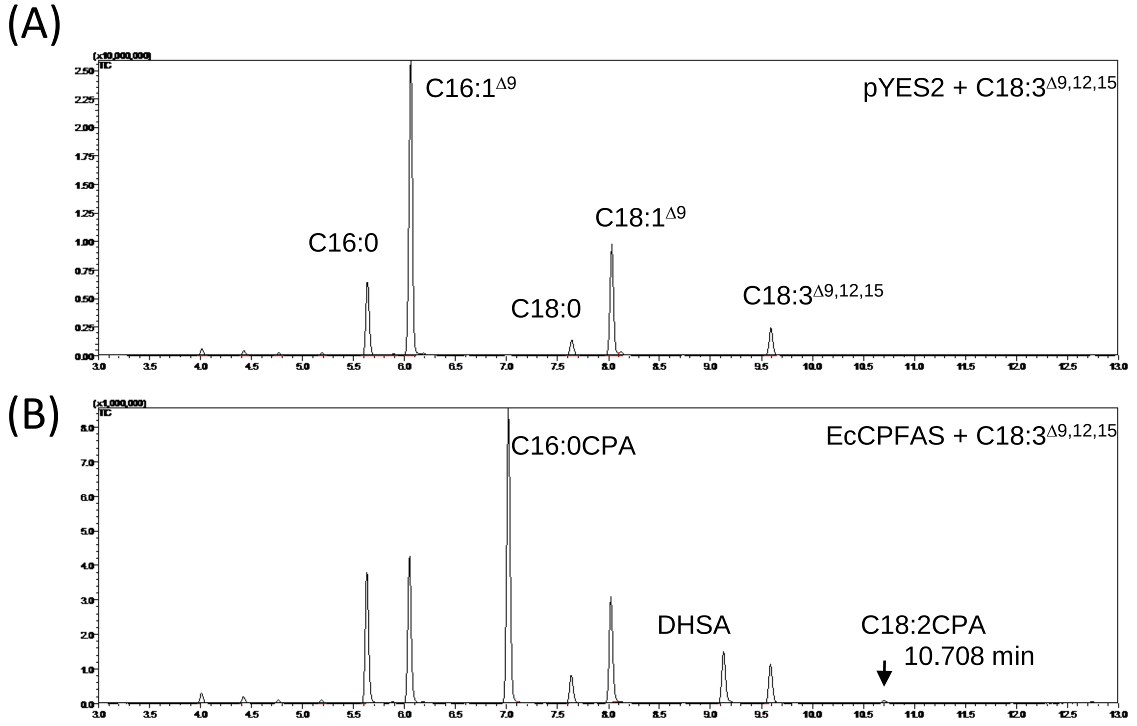

Supplementary Figure 1. Total fatty acid methyl ester (FAME) profile of *Saccharomyces cerevisiae* INVSc1 exogenously supplied with α-linolenic acid C18:3Δ9Z,12Z,15Z. (A) INVSc1 containing empty yeast expression vector pYES2, (B) INVSc1 containing *E. coli* cyclopropane fatty acid synthase (EcCPFAS).

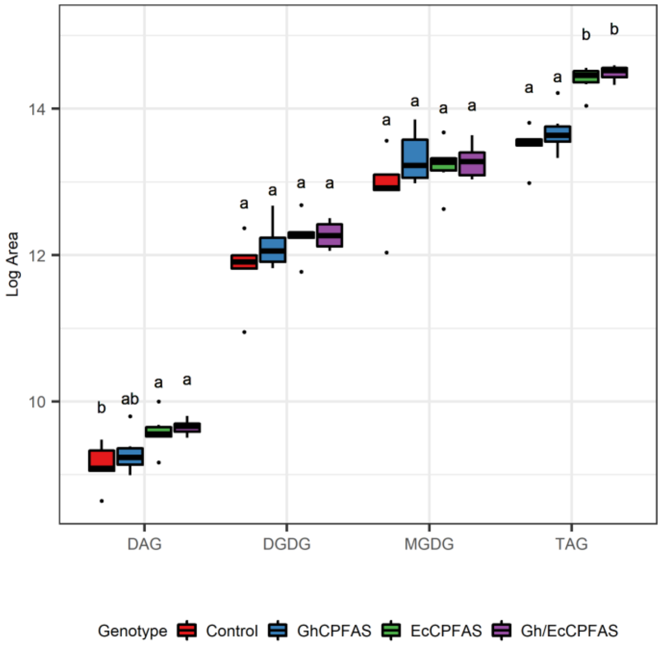

Supplementary Figure 2. Log peak areas of four lipid classes of *Nicotiana benthamiana* leaf expressing cotton and/or *E. coli* cyclopropane fatty acid synthase (CPFAS)) identified by liquid chromatography-tandem mass spectrometry (LC-MSMS). The control contained V2, GFP, and *hpNbFAD2.1*. Error bars are standard deviations of six replicates. Gh, cotton; Ec, *E. coli*; DAG, diacylglycerol; DGDG, digalactosyldiacylglycerol; MGDG, monogalactosyldiacylglycerol; TAG, triacylglycerol.

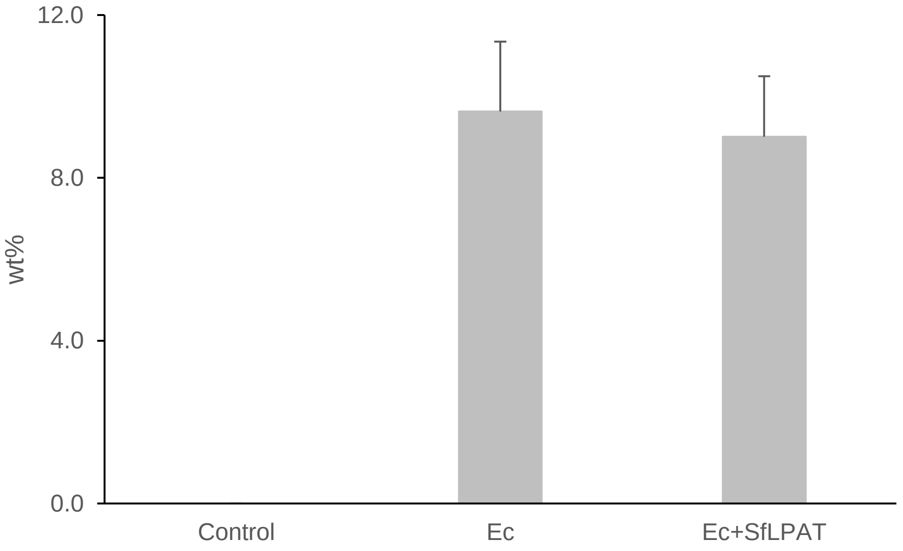

Supplementary Figure 3. Percentage of dihydrosterculic acid methyl ester in total fatty acid methyl ester of *Nicotiana benthamiana* leaf expressing *E. coli* cyclopropane fatty acid synthase (Ec) with or without *Stercullia foetida* lysophosphatidic acid acyltransferase (SfLPAT). The control contained V2, GFP, *hpNbFAD2.1*, and AtDGAT1. Error bars are standard deviations of six replicates. Ec, *E. coli* CPFAS; wt%, weight %.
